# Design of a Patient‐Specific 3D‐Printed Splint for Surgical Correction of Pectus Excavatum in a Kitten

**DOI:** 10.1155/crve/2686162

**Published:** 2026-04-24

**Authors:** Joana Reis, Kervin Ovalles, João Araújo, Fernando Silva, Rafael Coelho, Marco Gomes, José Simões

**Affiliations:** ^1^ CISAS, Escola Superior Agrária, Instituto Politécnico de Viana do Castelo, Viana do Castelo, Portugal, ipleiria.pt; ^2^ Centro de Bem Estar Animal-Clínica Veterinária, Escola Superior Agrária, Instituto Politécnico de Viana do Castelo, Viana do Castelo, Portugal, ipleiria.pt; ^3^ SEVN-Serviço de Ecografia Veterinária do Norte, Braga, Portugal; ^4^ Hospital Veterinário do Bom Jesus, Braga, Portugal; ^5^ ESAD.IDEA-Research in Design and Art, Matosinhos, Portugal

**Keywords:** 3D printing, kitten, pectus excavatum, splint, thoracic surgery

## Abstract

Pectus excavatum is a rare congenital deformation of the chest wall. Associated clinical signs depend on the severity of the deformity of the sternum and costochondral cartilages. After thoracic radiographic studies, a patient‐specific plastic external splint was designed based on a previously made plaster cast of the chest of the kitten. The splint was designed using 3D scan equipment and data processing to obtain the computer‐aided model that was then used to manufacture the splint using 3D printing technology. The surgical application of the customized 3D‐printed external splint for the correction of severe pectus excavatum in the young kitten was successful, with excellent tolerance observed postoperatively. After a period of 2 weeks, the splint was removed. Marked improvement of the sternum position was observed, and, more importantly, improvement in dyspnea and exercise intolerance was evident.

## 1. Introduction

Pectus excavatum (PE) means “hollow breast” in Latin and refers to a chest wall deformity seen frequently in humans [1, 2] and less often in veterinary patients [3, 4]. In veterinary patients, genetic predispositions are suspected but not fully elucidated [5, 6]. The etiopathogenesis of PE remains multifactorial, involving abnormal growth of the costal cartilage, genetic factors, and biomechanical alterations [7–9]. It is a congenital deformation of the thoracic wall, characterized in animals by a ventrodorsal depression. The altered alignment of the costal cartilages with the sternum is the cause of the deformation and cardiopulmonary compression [10]. This pathology, affecting from 1 human per 1000 up to 1 human per 400 live births, is rare in pets; however, it is more frequently observed in cats than in dogs [11, 12]. In both humans and animals, PE may compromise thoracic compliance, cardiac filling, and pulmonary function [5, 13, 14].

Treatment for PE depends on the severity of the clinical signs [15]. Milder cases have an indication for conservative treatment, and in severe cases, surgical treatment is indicated [10]. According to Singh et al. [16], physical therapy through medial compression of the thorax has been recommended to help strengthen the chest muscles and help develop the ribcage. However, conservative treatment of severely affected kittens is associated with a poorer prognosis [10, 17]. The surgical techniques of correction differ between humans and kittens. In humans, several techniques are used; the Ravitch technique (described in 1949 for the first time) is an open chest approach that involves partial resection of the cartilage, osteotomy of the sternum, xiphoid excision, and, sometimes, placement of a metal or allogenic bone strut for stabilization [18]; the Nuss procedure, described in 1998, is considered minimally invasive and relies on the placement of one or two transversal convex steel bars under the sternum, sutured in place to the thoracic wall muscles [19]. Recent refinements to the Nuss and Ravitch procedures, including video‐assisted and minimally invasive modifications, have improved outcomes and reduced recurrence [20, 21].

Other techniques have been described more recently, such as the cross‐bar [22]. [23]. [23] have introduced a medical device called the elastic bar, designed to correct PE. This technique takes advantage of a phenomenon observed in pectus carinatum patients, where prolonged application of force leads to reduced chest wall stiffness. By applying this principle, the elastic bar allows for the gradual correction of the deformity.

Recently, the treatment of pectus deformities has been shifting from traditional methods toward more customized solutions. This evolution is driven by the adoption of innovative rapid prototyping tools that enable the design and fabrication of patient‐specific treatments and medical devices [24]. In fact, personalized, computer‐aided, and 3D‐printed devices have revolutionized the correction of thoracic deformities [25, 26], with applications expanding to veterinary surgery [27–29].

Several authors have highlighted the clinical benefits of combining three‐dimensional reconstruction technology with 3D printing in the management of PE. In a study published by Gaspar Pérez et al. [30], the authors utilized 3D‐printed, customized Nuss bars and concluded that tailoring the shape and size of the bars facilitates surgical planning. These customized devices enable highly accurate and optimal morphological correction, reducing the likelihood of requiring removal or reinsertion and consequently lowering the risk of surgical complications. Shan et al. [31] report that this combined approach enhances the safety and effectiveness of thoracoscopic‐assisted Nuss procedures in humans, offering significant clinical value in treating PE. To address a PE defect that could not be reconstructed using conventional techniques, Cheng et al. [32] implanted a 3D‐printed, custom‐made, biodegradable, and highly porous scaffold filled with autologous fat graft. This approach resulted in an excellent aesthetic outcome, and the highly porous polycaprolactone implant was well tolerated by the patient.

Due to obvious differences in the conformation of the thorax of humans and cats, these solutions and techniques are not directly translatable, although surgical treatment may cause similar complications.

In cats, the severity of the deformity is traditionally graded using the vertebral index (VI) and frontosagittal index (FSI) as determined from orthogonal thoracic radiographs [17], and both external splint stabilization with circumsternal sutures [33–35] and internal splinting are described for correction of PE [36] and a combination of osteotomy and external splinting [37]. As in humans, the devices used may need customization. Mattioli et al. [38] used a customized 3D‐printed external splint for the correction of a severe PE in a 3‐month‐old kitten. These authors refer to this type of splint, associated with a progressive correction of the PE, as a better solution for the treatment of severe cases where a sudden distension of the rib cage could cause re‐expansion injuries.

## 2. Case Description

### 2.1. Clinical History and Presentation

A female European shorthair kitten, with an estimated age of 11–12 weeks, under the care of a rescue association, presented with a history of stunted growth, tachypnea, dyspnea, and a palpable deformation of the sternum, leading to a presumptive diagnosis of PE. At the primary care clinical practice, thoracic radiographs were taken, and FSI and VI were both calculated (VI = 4.55 and FSI = 3.34), grading the PE as severe. The abnormal positioning of the heart raised concerns about possible concomitant malformations. The kitten was referred for detailed diagnosis and treatment. Thoracic radiographs and abdominal ultrasonography were performed to exclude the presence of additional malformations and to determine FSI and VI with precision.

**Figure 1:**
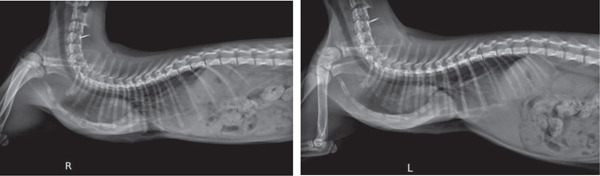
Severe pectus excavatum (laterolateral projections taken prior to external splint application).

**Figure 2:**
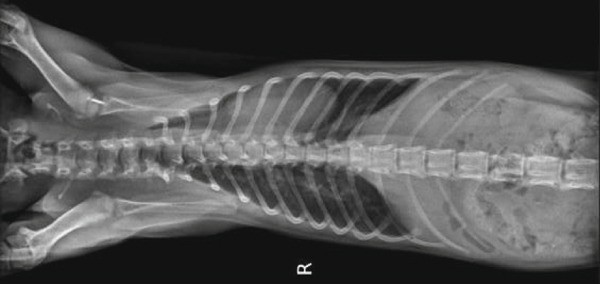
Severe pectus excavatum (radiograph taken prior to external splint application).

Imagiological exams confirmed the presence of severe PE (Figures 1 and 2), with pulmonary compression and the heart dislocated from its normal position.

The radiologic exams showed a pronounced displacement, from the central axis toward the left hemithorax, of the cardiac silhouette and of a portion of the mediastinum, occupying the space corresponding to both the cranial and caudal segments of the left pulmonary lobe. The FSI (FSI = 4.51) and the VI (VI = 2.33) were determined using lateral and ventrodorsal thoracic radiographic projections. The abdominal ultrasound confirmed the heart′s laterocaudal dislocation and absence of diaphragmatic defects or other abnormalities. An echocardiography was performed, but due to the abnormal position of the heart and small patient size, it was not possible to perform the plans needed for accurate measurements; however, the exam ruled out shunts or other cardiovascular congenital defects, and no cardiac remodelling was detected.

Given the severity of the deformation (considering FSI, PE is severe if FSI is > 3.0; considering VI, a value < 6 indicates severe PE) and its cardiovascular and respiratory consequences, surgical correction was recommended [17].

### 2.2. Splint Design and 3D Printing

The decision between using a commercially available, moldable splint and a custom 3D‐printed splint involves both clinical and financial considerations. Martelli et al. [39] conducted a systematic review of 3D printing applications in human medicine surgery, highlighting its benefits in preoperative planning, education, and the production of custom implants and anatomical models. They concluded, however, that the additional cost and time required to manufacture devices using current 3D printing technologies still limit their widespread adoption in clinical settings. The authors also emphasized the need for standardized guidelines to improve the reporting of surgical experiences involving 3D printing.

Commercial splints are readily available and generally offer a more affordable and immediate solution for routine orthopedic use. However, they often lack the anatomical precision needed to effectively treat complex or uncommon deformities, such as PE in small animal patients. In contrast, 3D‐printed splints require an upfront investment in imaging, design, and fabrication technology, which can increase initial costs. Despite this, their ability to be tailored precisely to a patient′s unique anatomy offers significant advantages such as superior fit, improved comfort, and potentially better clinical outcomes.

In the case presented, the 3D‐printed splint was designed using a combination of a plaster cast and a digital scan of the patient′s thoracic conformation. This approach resulted in a more accurate anatomical correction and resolution of clinical signs. While the production costs of 3D‐printed devices may be higher, their customization and precision can justify the expense, especially in cases where conventional splints prove inadequate or ineffective. The added investment may be offset by enhanced clinical results, reduced complications, and greater corrective accuracy.

**Figure 3:**
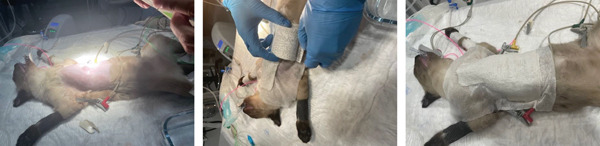
The plaster model making for surface geometry acquisition, splint design, and manufacturing.

For the design of the splint, a plaster model of the chest surface of the kitten was made. Figure 3 shows images of the plaster model making.

This model was then digitized using 3D scanning technology. The three‐dimensional scanning process was carried out using a structured light optical scanner (Heavy Duty Quadro from eviXscan 3D), which has an accuracy of less than 0.020 mm. The relevant geometry of the chest, a concave region, required multiple scans from different orientations to avoid shadowed areas. To facilitate this process and ensure automatic alignment of the captured images, an indexed rotary table was used.

The structured light technique involves projecting a pattern of lines onto the object. Two cameras were used to capture the reflected image, and subsequently, specific software was used to analyze the deformation of the patterns to reconstruct the three‐dimensional geometry. The result of this process was a point cloud, which in this specific case consisted of more than 3.5 million points. From this point cloud, a triangulated mesh with approximately 7 million triangles was generated and then exported in STL format.

**Figure 4:**
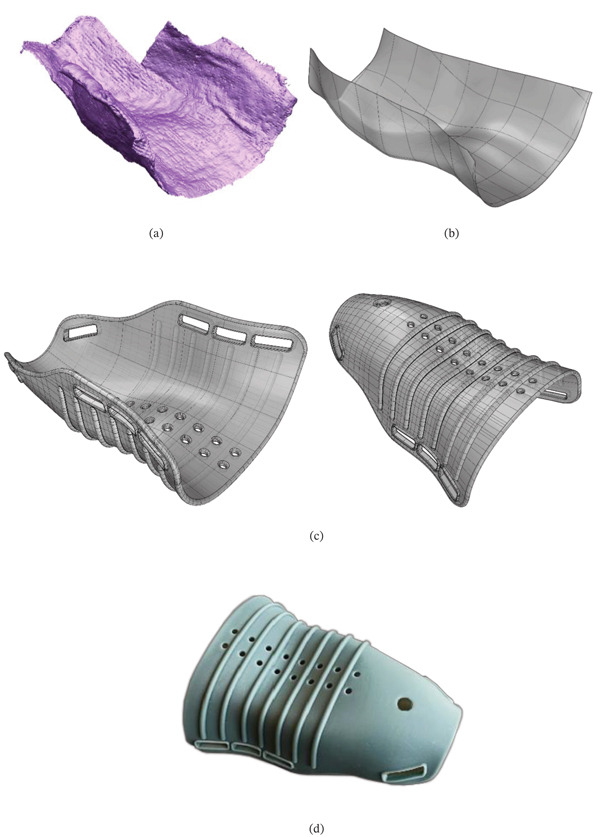
(a) Triangle mesh, (b) surface model, (c) CAD model, and (d) 3D‐printed splint.

The next step involved converting the triangle mesh into a surface model. For this purpose, the triangles were imported into Autodesk PowerShape Ultimate 2023 software. A best fit surface was automatically created over the mesh, with a defined tolerance of 0.1 mm. The triangle model was approximated to the inner surface of the plaster model, and a CAD model was generated with Rhinoceros software. This model was then used in a 3D printing equipment (Elegoo Mars) to print the splint that was used for the surgical procedure. A water‐washable resin was used, and the manufacturing time was around 7 h. This resin, known for its convenience and environmental protection, has characteristics of water solubility, low viscosity, and ultraviolet (UV) stability. The resin contains water‐soluble components such as polyvinyl alcohol or polycarboxylic acid that can be washed off with water [40]. UV was used to totally cure the plastic material of the splint. Figure 4 depicts images of the triangle model, surface model, CAD model, and 3D‐printed splint.

3D‐printed splints also have certain drawbacks. They involve higher initial costs, require access to reverse engineering tools and 3D printing equipment, and are more time‐consuming to produce compared to off‐the‐shelf options.

Table 1 presents the estimated costs for commercial splints and those manufactured using 3D printing. The cost of the splint developed and produced in this study was approximately €245. The surgical procedure costs were not included in the calculation. For the estimated cost presented in Table 1, a labor rate of €30/h was considered for obtaining the primitive geometry (triangle mesh and surface model) of the splint based on the plaster mold, as well as for preparing and modelling the digital design (CAD model) used for 3D printing the splint. However, labor rate varies widely among different countries, and this should be considered when planning.

**Table 1:** Estimated costs for commercial and 3D printing splints.

**Item**	**Estimated costs**	
Commercial splint or moldable plastic	€25–€70	
Padding and bandages	€10–€20	
Labor (fitting and monitoring)	€85–€130	
Total estimated cost	€120–€220	

**3D printing splints**		**Designed splint**
3D scan and imaging (CT or 3D surface scan)	€85–€250	€60 (including plaster mold)
CAD modelling	€40–€130	€60
3D printing (resin or filament and time)	€25–€80	€75
Labor (design, setup, and postprocessing)	€80–€180	€50
Total estimated cost	€230–€640	€245

Table 2 outlines the different characteristics of the two types of splints. As shown, material costs are relatively low. However, a biocompatible material should be placed between the splint and the animal′s chest to prevent skin reactions and other adverse effects.

**Table 2:** Characteristics between commercial and 3D‐printed splints.

	Commercial splint	3D‐printed splint
Overall cost	€120–€220	€230–€640
Material cost	Thermoplastic (low to moderate cost)	PLA, PETG, TPU, or medical‐grade resins (moderate cost)
Fit	Generic	Patient‐specific
Effectiveness (severe cases)	Limited	High
Labor expertise	Minimal	High (requires reverse engineering, CAD modelling, and technical skills)
Time to application	Immediate	1–2 days
Technological requirement	Low	High
Equipment cost	None or a heat gun	3D scanner (€250) and 3D printer (€300)
Reproducibility	Low–hard to replicate exactly	High‐digital files
Customization	Minimal	Complete
Clinical outcome (complex case)	Often suboptimal fit	Improved anatomical conformity and patient comfort
Training required	Basic clinical handling	CAD software + printer operation

Regarding the time required for the development and fabrication of the splint, digital manufacturing demands skilled professionals, which significantly increases the cost of this type of device. Currently, the cost of equipment (3D scanners and 3D printers) is no longer a major concern, as they are relatively affordable and offer excellent dimensional and geometric accuracy.

### 2.3. Surgery and Outcome

The kitten was placed under general anesthesia. Polypropylene–polyethylene monofilament nonabsorbable 2/0 sutures were used. The most caudal suture was placed around the xyphoid process, as close to the sternum as possible. The suture was elevated with gentle traction, and the next suture was placed cranially (seventh and sixth stenebrae).

**Figure 5:**
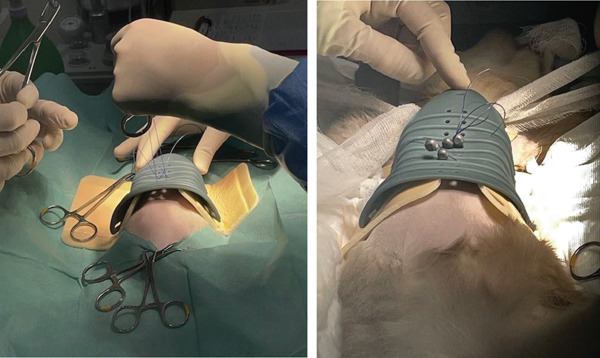
Surgical application of the external splint.

Gentle traction was applied, and each suture was kept long and held by a mosquito hemostat. Padding material was placed between the splint and the chest wall, except over the sternum, to increase comfort, prevent dermatitis, and add space between the splint and the chest, as seen in Figure 5. The end of each suture was passed through the holes in the splint, and traction was maintained, aiming at repositioning the sternum at a more ventral and physiological position. A total of four tension bands were applied, with sinkers to enable progressive tension adjustments.

The postoperative period was uneventful, with proper pain control ensured by a combination of opioids (methadone, followed by buprenorphine 2 days after surgery), dexmedetomidine (0.0125 mg/kg iv), meloxicam, and gabapentin. During the postoperative period, respiratory rates varied, with episodes of tachypnea but without dyspnea.

**Figure 6:**
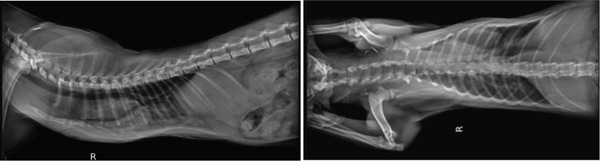
Radiographs taken immediately after the removal of the external splint, 13 days after its application.

Thirteen days after its application, the external splint was removed, as mild dermatitis had developed. Follow‐up thoracic radiographs were taken. The sternum position (Figure 6), albeit not completely corrected, was much closer to the physiological position. More importantly, the associated dyspnea and exercise intolerance were much improved, as well as food intake and weight gain (200 g in the following 2 weeks).

**Figure 7:**
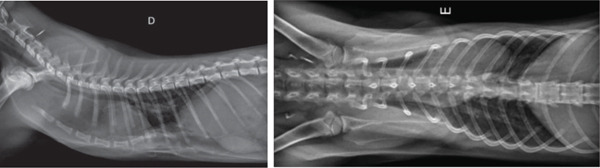
Radiographs taken before the placement of the second external splint.

Once the dermatitis had resolved, approximately 20 days after removing the 3D‐printed external splint, the kitten was submitted to a second intervention. A second external splint was placed under a similar technique for further positional correction of the sternum. Figure 7 shows radiographs taken prior to the second intervention.

**Figure 8:**
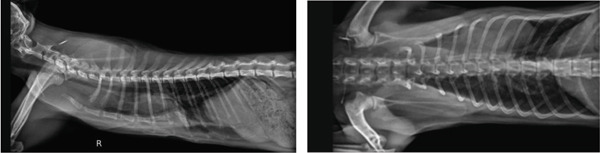
Radiographs taken after the removal of the second external splint.

The second splint remained in place for approximately 3 weeks. Radiographs were taken after its removal (Figure 8).

After considering the marked improvement of clinical signs previously observed and the fact that any further correction would likely entail invasive procedures, given that skeletal compliance had decreased with growth, a decision of not pursuing any attempts at further correction was made. Instead, clinical monitoring was elected.

**Figure 9:**
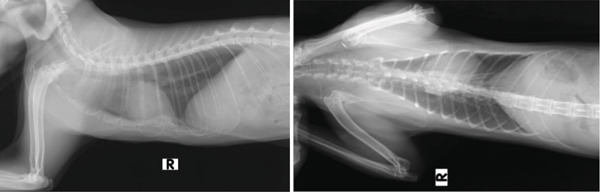
Follow‐up radiographs (September 11, 2025).

In September 2025, approximately 22 months after the removal of the second splint, a follow‐up appointment was scheduled at the primary clinical center. The tutor reported normal activity and appetite. The physical exam showed no abnormal findings apart from a localized sternum deformity, body condition was lean (4/9), and body weight was 3750 kg. The cat had been neutered the previous year without any complications, both during and after surgery. Thoracic radiographs (Figure 9) were taken, and FSI (1, 3) and VI (6, 12) were determined by the veterinary surgeon responsible for the radiographic diagnosis at the referral hospital.

## 3. Discussion

The splint was sufficiently robust to withstand the traction exerted by the suture threads and effectively supported the proper repositioning of the sternum. Although padding was added, the patient developed mild dermatitis, leading to a nonideal early removal of the splint and a 20‐day interruption in the correction of the deformity. Dermatitis is frequently associated with external splints and bandages [33, 34], but it was absent in the case report by Mattioli [38]. Due to kitten growth, which demanded a bigger external splint approximately 5 weeks after the first procedure, the surgeon opted for a plain external splint in the final procedure due to monetary and time limitations.

The radiographs in Figure 1 were taken during the first consultation at the referral center. However, the plaster cast was made 1 month later, and the 3D splint was applied 9 days after the plaster cast. These dates have implications for the age at which the patient was treated. According to the kitten′s estimated date of birth, the first splint (3D‐printed) was applied when the patient was approximately 18 weeks old. The success of external splinting depends on the pliability of the costal cartilage and sternum. Thus, this method is usually indicated for animals under 4 months of age [41]. In symptomatic cats older than 5 months presenting with a noncompliant sternum, more invasive surgical techniques are indicated, such as internal splinting [36, 41]. The sternum becomes less compliant in older kittens, although each case should be assessed individually [17]. Kudej [41] also states that external splints should be left in place between 2 and 3 weeks, although shorter placements may be successful. In the present clinical case, a longer total period was deemed necessary.

The resulting sternal alignment, even with xyphoid luxation, restored cardiopulmonary function. The present FSI and VI are borderline but normal.

Horn et al. [42] reported the use of an external splint made from polyurethane, with an internal neoprene lining, in the surgical repair of PE in a young feline. Unlike our approach, which utilized a plaster mold, their process involved the use of CT imaging to shape the 3D‐printed splint. The authors noted that this method enabled improved surgical planning, ultimately enhancing patient comfort.

Conventional commercially available moldable splints are widely accessible, cost‐effective, and easy to use, as they do not require advanced imaging or customized design processes. However, they have several limitations, including poor anatomical fit for complex deformities, a higher risk of pressure sores or dermatitis, the need for frequent adjustments or replacements, and reduced effectiveness in severe cases [38, 42].

In contrast, 3D‐printed custom splints offer a tailored anatomical fit, which improves patient comfort and correction outcomes, particularly in severe or complex deformities [38, 42]. Additionally, the digital model used for their creation can be employed in computational simulations, such as finite element analysis, to optimize design and performance.

In terms of cost, commercial moldable splints can be two to four times more expensive. For routine cases, moldable splints are generally preferred due to their simplicity and ease of use. However, in cases involving complex deformities, 3D‐printed splints are often clinically and economically justified, offering improved outcomes and the added benefit of reusable digital designs.

## 4. Conclusions

This case demonstrates the successful application of a customized 3D‐printed external splint for the surgical correction of severe PE in a young kitten. The individualized approach, involving detailed imaging, digital modelling, and precise fabrication, allowed for superior anatomical conformity and effective repositioning of the sternum. Despite minor complications such as dermatitis, the use of the 3D‐printed splint led to significant clinical improvement, including reduced dyspnea and improved thoracic conformation. This case supports the potential benefits of personalized medical devices in veterinary surgery, particularly for complex or rare deformities where traditional methods may be inadequate. While initial costs and technical requirements may be higher, the improved outcomes and tailored fit justify the investment in 3D printing technologies for selected cases in small animal practice.

## Funding

This work is funded by national funds through FCT‐Fundação para a Ciência e a Tecnologia, I.P., under the Projects UID/05237/2025 (https://doi.org/10.54499/UID/05237/2025) and UID/05937/2025 (https://doi.org/10.54499/UID/05937/2025).

## Consent

The owner of the animal has given authorization for the publication of this report.

## Conflicts of Interest

The authors declare no conflicts of interest.

## Data Availability

The data that support the findings of this study are available upon request from the corresponding author. The data are not publicly available due to privacy or ethical restrictions.
